# Acute Disruption of the Dorsal Hippocampus Impairs the Encoding and Retrieval of Trace Fear Memories

**DOI:** 10.3389/fnbeh.2019.00116

**Published:** 2019-05-29

**Authors:** Jacob H. Wilmot, Kyle Puhger, Brian J. Wiltgen

**Affiliations:** ^1^Department of Psychology, University of California, Davis, Davis, CA, United States; ^2^Center for Neuroscience, University of California, Davis, Davis, CA, United States

**Keywords:** learning, memory, optogenetics, context fear, mice

## Abstract

A major function of the hippocampus is to link discontiguous events in memory. This process can be studied in animals using Pavlovian trace conditioning, a procedure where the conditional stimulus (CS) and unconditional stimulus (US) are separated in time. While the majority of studies have found that trace conditioning requires the dorsal segment of the hippocampus, others have not. This variability could be due to the use of lesion and pharmacological techniques, which lack cell specificity and temporal precision. More recent studies using optogenetic tools find that trace fear acquisition is disrupted by decreases in dorsal CA1 (dCA1) activity while increases lead to learning enhancements. However, comparing these results is difficult given that some studies manipulated the activity of CA1 pyramidal neurons directly and others did so indirectly (e.g., *via* stimulation of entorhinal cortex inputs). The goal of the current experiments, therefore, was to compare the effects of direct CA1 excitation and inhibition on the encoding and expression of trace fear memories. Our data indicates that stimulation of ArchT in dCA1 pyramidal neurons reduces activity and impairs both the acquisition and retrieval of trace fear. Unlike previous work, direct stimulation of CA1 with ChR2 increases activity and produces deficits in trace fear learning and expression. We hypothesize that this is due to the artificial nature of optogenetic stimulation, which could disrupt processing throughout the hippocampus and in downstream structures.

## Introduction

The hippocampus integrates spatial and temporal information to form complex memory representations. These include episodic memories in humans and contextual memories in animals (Eichenbaum, 2017). Simple associations, in contrast, can typically be learned without this structure. For example, rodents with damage to the hippocampus can acquire fear to an auditory cue that is immediately followed by shock (Chowdhury et al., 2005; Esclassan et al., 2009). However, if the shock is presented several seconds after the cue has ended, the same animals cannot form an association between them. This suggests that an important function of the hippocampus is to link discontiguous events—a property that allows it to encode sequences and form spatial maps, both of which involve associations between stimuli that are separated in time.

The ability to learn temporal associations can be studied in animals using trace conditioning. This is a Pavlovian procedure where a gap is inserted between the termination of the conditional stimulus (CS) and the onset of the unconditional stimulus (US). The majority of studies have found that the acquisition and retrieval of trace conditioning require the dorsal hippocampus (Chowdhury et al., 2005; Raybuck and Lattal, 2011, for review Raybuck and Lattal, 2014) although there are exceptions (Yoon and Otto, 2007; Czerniawski et al., 2009; Cox et al., 2013). This variability could be attributed to the use of lesion and pharmacological techniques, both of which lack cell specificity and temporal precision. More recent studies have utilized optogenetic tools to directly manipulate hippocampal neurons or alter their activity indirectly by stimulating entorhinal inputs. When CA1 activity was decreased during learning, deficits in trace fear conditioning were observed (Kitamura et al., 2014). In contrast, activation of CA1 neurons enhanced learning in young mice and ameliorated aging deficits in older animals (Kitamura et al., 2014; Sellami et al., 2017).

The goal of the current study was to directly compare the effects of CA1 stimulation on the acquisition and retrieval of trace fear memories. Based on previous work, we predicted that activation of dorsal CA1 (dCA1) pyramidal neurons would enhance learning while inhibition would impair both encoding and retrieval. The effect of CA1 activation on memory expression was less clear. Although it is possible to drive the retrieval of contextual fear memories by stimulating neurons in the dentate gyrus (Liu et al., 2012), the same procedure is far less effective in CA1 (Ramirez et al., 2013; Ryan et al., 2015). In addition, optogenetic activation of ventral CA1 has been shown to impair the retrieval of contextual fear (Jimenez et al., 2018). Accordingly, we predicted that direct stimulation of dCA1 neurons would either impair or have no effect on the expression of trace fear.

## Materials and Methods

### Subjects

Subjects in this study were 2–4-month-old male and female C57BL/6J mice (Jackson Labs). Mice were maintained on a 12 h light/12 h dark cycle with *ad libitum* access to food and water. All experiments were performed during the light portion (7 a.m–7 p.m.) of the light/dark cycle. Mice were group housed until surgery, at which point they were single housed for the rest of the experiment.

### Surgery

Stereotaxic surgery was performed 2–3 weeks before behavioral experiments began. Mice were anesthetized with isoflurane (5% induction, 2% maintenance) and placed into a stereotaxic frame (Kopf Instruments). An incision was made in the scalp and the skull was adjusted to place bregma and lambda in the same horizontal plane. Small craniotomies were made above the desired injection site in each hemisphere. AAV was delivered at a rate of 2 nl/s to dCA1 (AP −2.0 mm and ML ±1.5 mm from bregma; DV −1.25 mm from dura) through a glass pipette using a microsyringe pump (UMP3, World Precision Instruments). For stimulation experiments, the AAVs used were AAV9-CaMKIIa-hChR2(H134R)-eYFP (250 nl/hemisphere, titer: 8.96 × 10^13^, Penn Vector Core) and AAV9-CaMKIIa-eGFP (250 nl/hemisphere, titer: 3.49 × 10^13^, Penn Vector Core). For inhibition experiments, the constructs were AAV5-CaMKIIa-ArchT-GFP (350 nl/hemisphere, titer: 5.2 × 10^12^, UNC Vector Core) and AAV5-CaMKIIa-GFP (350 nl/hemisphere, titer: 5.3 × 10^12^, UNC Vector Core). After AAV infusions, an optical fiber (200 μm diameter, Thorlabs) was implanted above dCA1 in each hemisphere (AP −2.0 mm and ML ±1.5 mm from bregma; DV −1.0 mm from dura). The fiber implants were secured to the skull using dental adhesive (C&B Metabond, Parkell) and dental acrylic (Bosworth Company).

### Apparatus

The behavioral apparatus has been described previously (Tayler et al., 2011). Briefly, fear conditioning occurred in a conditioning chamber (30.5 cm × 24.1 cm × 21.0 cm) within a sound-attenuating box (Med Associates). The chamber consisted of a front-mounted scanning charge-coupled device video camera, stainless steel grid floor, a stainless steel drop pan, and overhead LED lighting capable of providing broad spectrum and infrared light. For context A, the conditioning chamber was lit with both broad spectrum and infrared light and scented with 95% ethanol. For context B, a smooth white plastic insert was placed over the grid floor and a curved white wall was inserted into the chamber. Additionally, the room lights were changed to red light, only infrared lighting was present in the conditioning chamber, and the chamber was cleaned and scented with disinfectant wipes (PDI Sani-Cloth Plus). In both contexts, background noise (65 dB) was generated with a fan in the chamber and HEPA filter in the room.

### Trace Fear Conditioning Procedure

All behavioral testing occurred during the light portion of the light/dark cycle. Mice were habituated to handling and optical fiber connection for 5 min/day for 5 days before the beginning of behavior. Then, the mice were habituated to context B with one 5-min session of free exploration each day for 2 days. Next, the mice underwent trace fear conditioning in context A. During training, mice were allowed to explore the conditioning chamber for 3 min before receiving six conditioning trials. Each trial consisted of a 20 s pure tone (85 dB, 3,000 Hz) and a 2 s shock (0.9 mA) separated by a 20 s stimulus-free trace interval. The intertrial interval (ITI) was 120 s. Mice were removed from the chamber 120 s after the last trial. Twenty-four hours later, the mice were placed in context B for a tone test consisting of a 3 min baseline period followed by six 20-s tone presentations separated by a 140 s ITI. Freezing behavior was used to index fear and measured automatically using VideoFreeze software (Med Associates). The next day, mice were placed back in the original conditioning chamber (context A) for either a 12- or 20-min context test, depending on the experiment.

### Experiment-Specific Methods

#### Experiment 1-ArchT Inhibition During Fear Memory Retrieval

Continuous green light (531 nm, 12 mW at fiber tip) illumination was delivered to dCA1 of mice expressing ArchT (*n* = 6) or eYFP (*n* = 6) during the tone and context testing periods. In the tone test, light onset was simultaneous with tone onset and lasted 40 s. The context test was 20 min and green light was delivered throughout the test in order to ensure c-Fos expression would be representative of neural activity that occurred while the laser was on.

#### Experiment 2-ChR2 Stimulation During Fear Memory Retrieval

Blue light (465 nm, 12 mW measured at fiber tip) was delivered (20 Hz, 15 ms pulse width) to dCA1 of mice expressing ChR2 (*n* = 5) or eGFP (*n* = 4) during the tone test and the context test. In the tone test, light onset was simultaneous with tone onset and lasted 40 s. The context test consisted of four 3-min epochs. The light was off for the first 3 min and on for the next 3 min; then, this sequence was repeated one time. Mice were sacrificed 90 min following the end of the context test in order to quantify c-Fos expression.

#### Experiment 3-ChR2 Stimulation During Trace Fear Encoding

As in Experiment 2, blue light was delivered to dCA1 of ChR2 (*n* = 6) and eGFP (*n* = 6) mice in 42 s epochs during the training session. Light onset was simultaneous with onset of the tone and the light coterminated with the shock. No light was delivered during the tone or context tests. The context test was 20 min.

#### Experiment 4-ArchT Inhibition During Trace Fear Encoding

As in Experiment 1, green light illumination was delivered to dCA1 of ArchT (*n* = 5) and eYFP (*n* = 7) mice during training in the same 42 s epochs described for Experiment 3. Light was not present during testing and the context test was 20 min.

### Immunohistochemistry

Ninety minutes after behavioral testing, mice were transcardially perfused with 4% PFA. Following 24 h of post-fixation, 40 μm coronal sections were cut and stained for c-Fos. Slices were washed three times in 1× phosphate buffered saline (PBS) at the beginning of the procedure and after all antibody and counterstaining steps. All antibodies and counterstains were diluted in a blocking solution containing 0.2% Triton-X and 2% normal donkey serum in 1× PBS, unless otherwise indicated. First, sections were incubated for 15 min in the blocking solution. Then, slices were incubated for 24 h at 4° in anti-c-Fos rabbit primary antibody (1:5,000, ABE457, Millipore). Next, slices were placed in biotinylated donkey anti-rabbit secondary antibody (1:500, Jackson ImmunoResearch) for 60 min at room temperature, followed by Streptavidin-Cy3 (1:500, Jackson ImmunoResearch) for 45 min. Finally, sections were stained with DAPI (1:10,000 in PBS, Life Technologies) for 10 min, mounted on slides, and coverslipped with Vectashield anti-fade mounting media (Vector Labs).

### Image Acquisition and Cell Quantification

Images were acquired at 20× magnification using a fluorescence slide scanner (BX61VS, Olympus). After acquisition, images were cropped to contain approximately 30,000–40,000 μm^2^ of dCA1. A blinded experimenter performed cell counts on 3–4 sections from each animal (6–8 hemispheres). c-Fos+ cells were counted using the multi-point tool in ImageJ. Cell counts were averaged across slices to obtain one value per animal.

### Statistical Analysis

For analysis of behavioral data from training and tone test sessions, freezing scores in each phase type (baseline, tone, trace) were averaged for each animal. All behavioral data were analyzed using two-way repeated-measures ANOVA followed by Bonferroni-corrected *post hoc* comparisons when necessary. Cell count data was converted to a percent change score for each animal. Percent control was calculated by first dividing the number of c-fos+ cells/mm^2^ in each animal by the mean number of c-fos+ cells/mm^2^ in the control group. These values were then multiplied by 100 to convert them to a percent change. These scores were analyzed using unpaired *t*-tests. A threshold of *p* < 0.05 was used to determine statistical significance. All data are shown as mean ± SEM. All data were analyzed with GraphPad Prism (v8) and all figures were generated using Prism and BioRender.

## Results

### Inhibition of dCA1 Impairs Trace Fear Memory Retrieval

**Figure 1:**
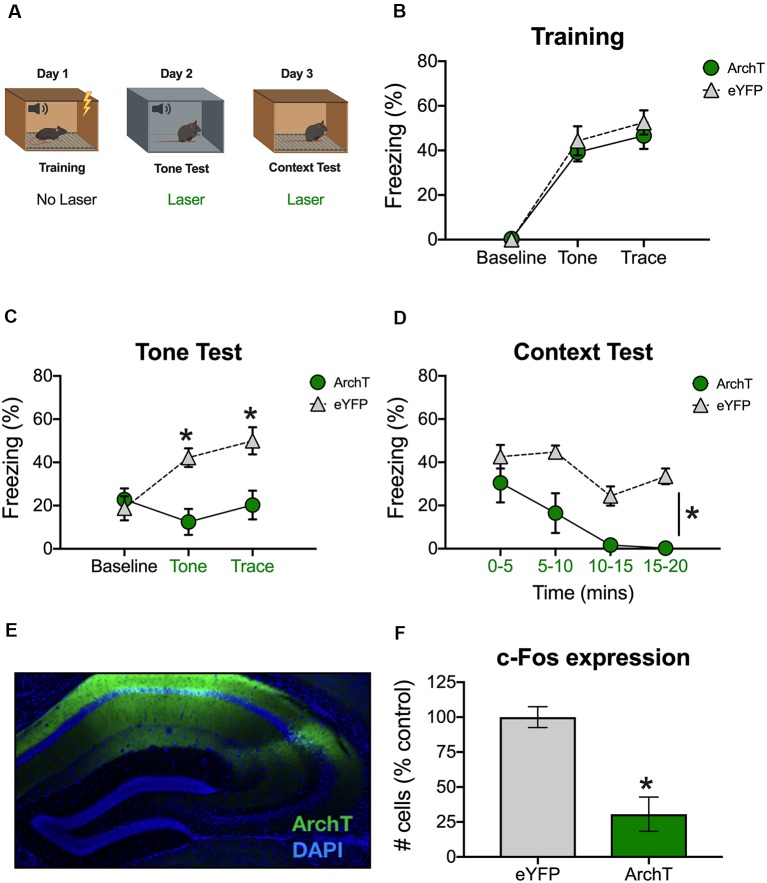
Inhibition of dCA1 impairs trace fear memory retrieval. **(A)** Schematic of behavioral paradigm. On day 1, animals underwent trace fear conditioning without laser stimulation. The next day, mice underwent a tone memory test in a novel context with green light delivered to dorsal CA1 (dCA1) during each trial. Twenty-four hours later, mice were placed back in the conditioning environment for a context memory test. Green light was delivered to dCA1 during the entire context test. **(B)** Freezing during the training phase of trace fear conditioning (Mean ± SEM). **(C)** Freezing during the tone test (Mean ± SEM). **(D)** Freezing during the context test (Mean ± SEM). **(E)** Example of virus expression. Green, ArchT; Blue, DAPI. **(F)** c-Fos expression in eGFP and ArchT mice after the context test. Green x-axis labels denote periods during which the laser was delivered. In all panels, green represents the ArchT group and gray represents the control group. *p < 0.05 relative to control.

To silence dCA1 during retrieval, we expressed the inhibitory opsin ArchT in pyramidal neurons using the αCaMKII promoter. Animals then received six trace fear conditioning trials in the absence of laser stimulation (Figure 1A). Each trial consisted of a 20-s auditory CS followed by a 20-s trace interval and then a 2 s footshock. The ITI was 120 s. As expected, freezing increased during the tone and trace interval relative to the baseline period and there were no differences between ArchT mice and eYFP control animals (Main effect of stimulus period *F*_(2,20)_ = 122, *p* < 0.05; No effect of group, *F*_(1,10)_ = 0.48, *p* > 0.05, No stimulus period × group interaction *F*_(2,20)_ = 0.49, *p* > 0.05; Figure 1B).

The next day, animals received a tone test in a novel environment. The test was identical to training except that no shocks were presented and continuous green light was delivered to dCA1 during the tone and trace intervals (Figure 1A). Group differences were not observed at baseline (BL); however, ArchT stimulation significantly reduced freezing during the tone and trace intervals [Group × stimulus period interaction *F*_(2,20)_ = 10.9, *p* < 0.05; Bonferroni *post hoc* tests, BL (*p* > 0.05), tone and trace (*p* < 0.05; Figure 1C)]. The following day, mice were placed back in the original training environment for 20-min to assess context fear. Continuous green light was delivered to dCA1 during the entire test (Figure 1A). Similar to the trace fear data, stimulation of ArchT significantly reduced freezing to the context (Main effect of group *F*_(1,10)_ = 23.81, *p* < 0.05; Main effect of time *F*_(3,30)_ = 10.48, *p* < 0.05; No group × time interaction *F*_(3,30)_ = 1.73, *p* > 0.05; Figure 1D).

To confirm that ArchT was expressed in dCA1 and that laser stimulation reduced neural activity, mice were sacrificed 90-min after the context test. We observed strong bilateral expression of ArchT and eYFP throughout the dCA1 (Figure 1E). We also found reduced expression of the immediate early gene c-Fos in ArchT mice relative to eYFP controls, indicating that our manipulation successfully reduced neural activity (*t*_(10)_ = 4.83, *p* < 0.05; Figure 1F). Together, these data demonstrate that reduced activity in dCA1 impairs the retrieval of both trace and context fear memories.

### Stimulation of dCA1 Impairs Trace Fear Memory Retrieval

**Figure 2:**
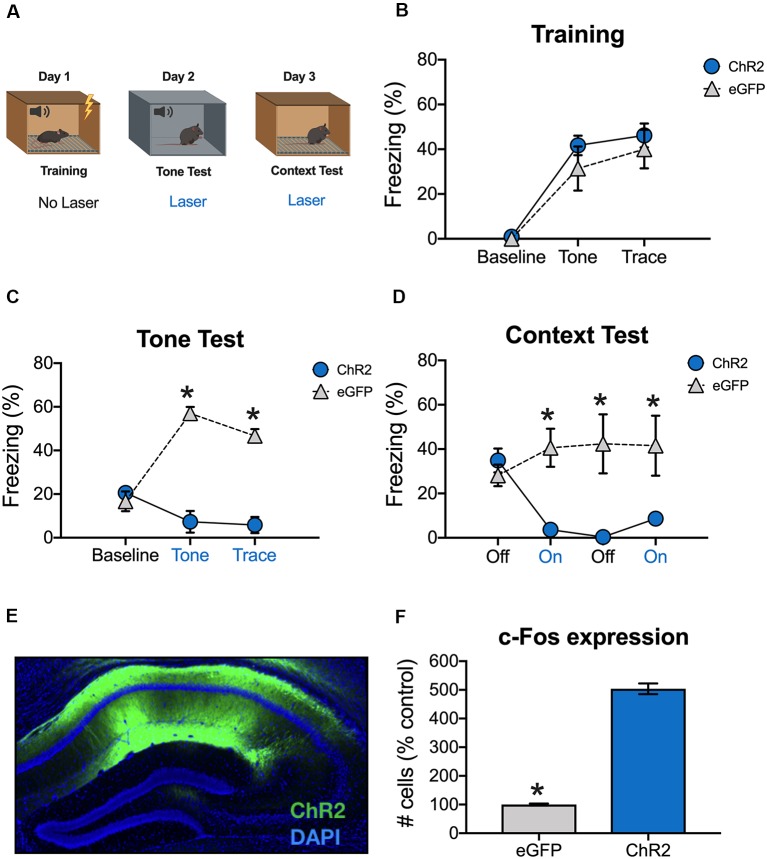
Stimulation of dCA1 impairs trace fear memory retrieval. **(A)** Schematic of behavioral paradigm. On day 1, animals underwent trace fear conditioning without laser stimulation. The next day, mice underwent a tone memory test in a novel context with blue light delivered (20 Hz) to dCA1 during each trial. Twenty-four hours later, mice were placed back in the conditioning environment for a context memory test. The laser was not turned on for the first 3 min of the context test. Then, blue light was delivered to dCA1 for the next 3 min, followed by another 3 min laser off period, and a last 3-min laser on epoch. **(B)** Freezing during the training phase of trace fear conditioning (Mean ± SEM). **(C)** Freezing during the tone test (Mean ± SEM). **(D)** Freezing during the context test (Mean ± SEM). **(E)** Example of virus expression. Green, ChR2; Blue, DAPI. **(F)** c-Fos expression in eGFP and ChR2 mice after the context test. Blue x-axis labels denote periods during which the laser was delivered. In all panels, blue represents the ChR2 group and gray represents the control group. *p < 0.05 relative to control.

To examine the effects of dCA1 stimulation on retrieval, we expressed the excitatory opsin ChR2 in pyramidal neurons under control of the αCaMKII promoter. Animals were trained and tested using the same procedure described in the previous experiment (Figure 2A). During training, freezing increased during the tone and trace intervals relative to the baseline period and no differences were observed between ChR2 and eGFP groups (Main effect of stimulus period *F*_(2,14)_ = 59.71, *p* < 0.05; No effect of group, *F*_(1,7)_ = 0.82, *p* > 0.05, No stimulus period × group interaction *F*_(2,14)_ = 0.63, *p* > 0.05; Figure 2B).

Animals received a tone test the next day, during which blue light (20 Hz) was delivered to dCA1 during the tone and trace intervals (Figure 2A). There were no group differences at baseline, but ChR2 stimulation significantly reduced freezing during the subsequent tone and trace intervals [Group × stimulus period interaction *F*_(2,14)_ = 43.7, *p* < 0.05; Bonferroni *post hoc* tests, BL (*p* > 0.05), tone and trace (*p* < 0.05; Figure 2C)]. Twenty-four hours later, the mice were put back in the original training environment to assess context fear. This test began with a 3-min laser off period (BL) followed by 3-min of blue light stimulation and 3-min of no stimulation. It ended with a second 3-min period of blue light stimulation. During BL, the groups froze at similar levels indicating that both had acquired context fear memories. However, when dCA1 was stimulated, freezing was significantly reduced in ChR2 mice relative to eGFP controls. Freezing remained low in this group after the laser turned off and did not recover for the remainder of the test session [Group × stimulus period interaction *F*_(3,21)_ = 12.34, *p* < 0.05; Bonferroni *post hoc* tests, BL (*p* > 0.05) all subsequent laser on and laser off periods (*p* < 0.05; Figure 2D)].

To examine virus expression and determine the effects of dCA1 stimulation on neural activity, mice were perfused 90 min after the context test. As expected, we observed robust expression of ChR2 (Figure 2E) and stimulation produced a large increase in the number c-Fos positive dCA1 neurons relative to eGFP controls (*t*_(7)_ = 18.78, *p* < 0.05; Figure 2F). These data demonstrate that stimulation of dCA1 neurons impairs the retrieval of both trace and context fear memories.

### Stimulation of dCA1 Impairs the Acquisition of Trace Fear Conditioning

**Figure 3:**
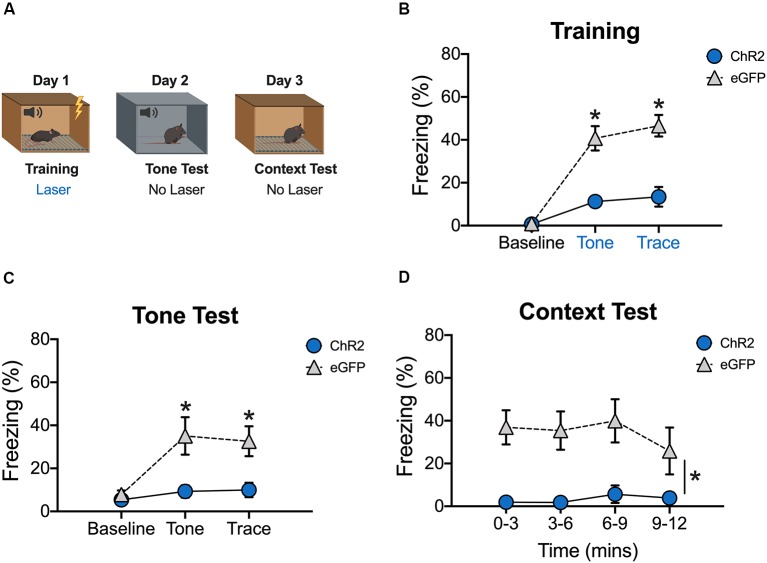
Stimulation of dCA1 during trace fear encoding impairs memory acquisition. **(A)** Schematic of behavioral paradigm. On day 1, animals underwent trace fear conditioning with blue light (20 Hz) delivered to dCA1 during each training trial. The next day, mice underwent a tone memory test in a novel context with no laser stimulation. Twenty-four hours later, mice were placed back in the conditioning environment for a context memory test without light delivery. **(B)** Freezing during the training phase of trace fear conditioning (Mean ± SEM). **(C)** Freezing during the tone test (Mean ± SEM). **(D)** Freezing during the context test (Mean ± SEM). Blue x-axis labels denote periods during which the laser was delivered. In all behavioral panels, blue represents the ChR2 group and gray represents the control group. *p < 0.05 relative to control.

We next determined the effects of stimulation on encoding by delivering blue light to dCA1 during each training trial (tone-trace interval-shock; Figure 3A). There were no group differences during the baseline period, but ChR2 stimulation significantly reduced freezing during the tone and trace intervals [Group × stimulus period interaction *F*_(2,20)_ = 18.2, *p* < 0.05; Bonferroni *post hoc* tests, BL (*p* > 0.05), tone and trace (*p* < 0.05; Figure 3B)]. The same effects were observed the next day when mice received a tone test in the absence of blue light stimulation [Group × stimulus period interaction *F*_(2,20)_ = 8.09, *p* < 0.05; Bonferroni *post hoc* tests, BL (*p* > 0.05), tone and trace (*p* < 0.05; Figure 3C)]. Twenty-four hours after the tone test, context memory was assessed by returning the mice to the training context. Blue light was not delivered during this session. Similar to the tone test data, context fear was significantly reduced in ChR2 mice relative to eGFP controls (Main effect of group *F*_(1,10)_ = 14.52, *p* < 0.05; Main effect of time *F*_(3,30)_ = 1.07, *p* < 0.05; No group × time interaction *F*_(3,30)_ = 0.96, *p* > 0.05; Figure 3D). Together, these data demonstrate that both trace and context fear memories are disrupted when dCA1 is stimulated during encoding.

### Inhibition of dCA1 Impairs the Acquisition of Trace Fear Conditioning

**Figure 4:**
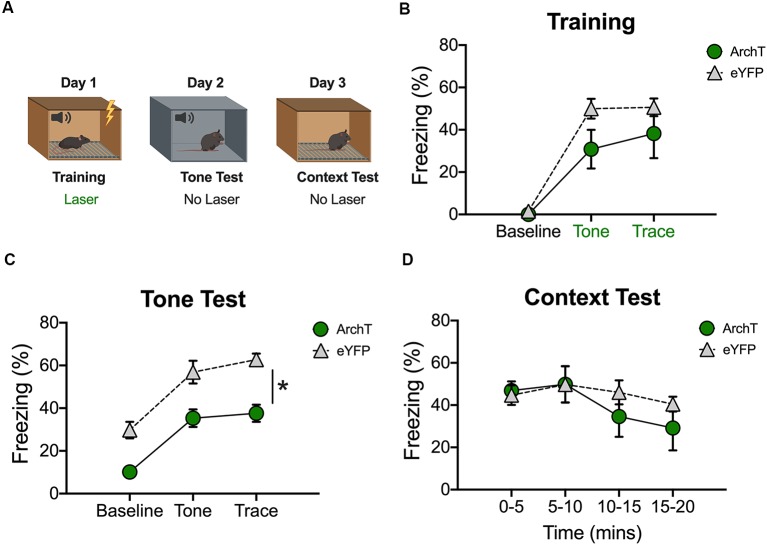
Inhibition of dCA1 during trace fear encoding impairs memory acquisition. **(A)** Schematic of behavioral paradigm. On day 1, animals underwent trace fear conditioning with green light delivered to dCA1 during each training trial. The next day, mice underwent a tone memory test in a novel context with no laser stimulation. Twenty-four hours later, mice were placed back in the conditioning environment for a context memory test without light delivery. **(B)** Freezing during the training phase of trace fear conditioning (Mean ± SEM). **(C)** Freezing during the tone test (Mean ± SEM). **(D)** Freezing during the context test (Mean ± SEM). In all behavioral panels, green represents the ArchT group and gray represents the control group. Green x-axis labels denote periods during which the laser was delivered. *p < 0.05 relative to control.

In our last experiment, we examined the effects of inhibition on trace fear encoding by stimulating ArchT during training (Figure 4A). As in the previous experiment, light was delivered to dCA1 during each conditioning trial (tone-trace interval-shock). Surprisingly, there were no differences between the ArchT and eYFP groups during the baseline period or during the tone and trace intervals (No effect of group *F*_(1,10)_ = 2.77, *p* > 0.05; Main effect of stimulus period *F*_(2,20)_ = 60.7, *p* < 0.05; No Group × stimulus period interaction *F*_(2,20)_ = 2.07, *p* > 0.05; Figure 4B). However, when memory was tested the next day (in the absence of light stimulation) ArchT animals froze significantly less than eYFP controls during all stimulus periods (Main effect of group *F*_(1,10)_ = 29.74, *p* < 0.05; Main effect of stimulus period *F*_(2,20)_ = 41.33, *p* < 0.05; No Group × stimulus period interaction *F*_(2,20)_ = 0.29, *p* > 0.05; Figure 4C). Twenty-four hours after the tone test, context memory was assessed by returning the mice to the training environment. Green light was not delivered during this session. The ArchT and eYFP groups froze at similar levels during this test indicating that dCA1 inhibition did not affect the formation of a context fear memory (No effect of group *F*_(1,10)_ = 0.53, *p* > 0.05; No effect of time *F*_(3,30)_ = 2.41, *p* > 0.05; No group × time interaction *F*_(3,30)_ = 0.74, *p* > 0.05; Figure 4D).

These data are consistent with a recent report and suggest that reduced activity in dCA1 disrupts the acquisition of trace but not context fear memories (Sellami et al., 2017).

### Altering dCA1 Activity Does Not Increase Exploration or Reduce the Response to Shock

**Figure 5:**
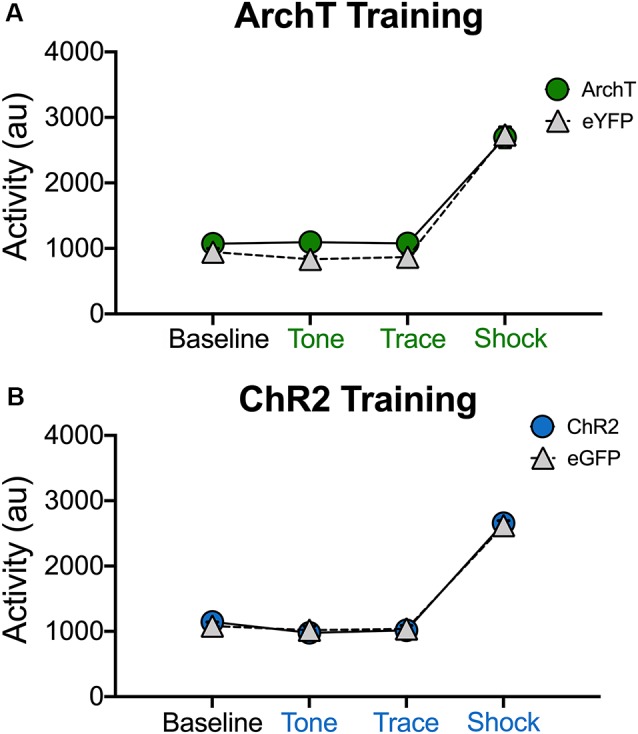
Stimulation and inhibition of dCA1 do not alter locomotor activity or shock responsivity. **(A)** Average motion (arbitrary units) during the last 20 s of baseline and the first tone, trace, and shock periods in Experiment 3 (inhibition during trace fear encoding; Mean ± SEM). **(B)** Average motion during the last 20 s of baseline and the first tone, trace, and shock periods in Experiment 4 (stimulation during trace fear encoding; Mean ± SEM).

It is possible that our manipulations impaired trace fear conditioning because they induced hyperactivity or disrupted the animals’ ability to process shock. This is unlikely given that optogenetic inhibition of dCA1 does not impair delay fear conditioning or increase activity in the open field (Goshen et al., 2011). In addition, optogenetic activation of dCA1 increases the ability of aged mice to acquire trace fear conditioning (Sellami et al., 2017). Nonetheless, we addressed this issue by determining if laser stimulation altered exploration or shock reactivity during the first conditioning trial (Figure 5). Only the first trial was analyzed because mice were exploring naturally and had not yet started freezing. In addition, endogenous opiates are released during fear conditioning and have been shown to reduce shock sensitivity (Fanselow and Bolles, 1979; Fanselow and Baackes, 1982). We quantified activity levels immediately before laser stimulation (BL) and then compared these to subsequent periods when the laser was on (tone, trace interval and shock). Analysis of our ArchT data revealed that activity levels were not altered when dCA1 was inhibited during the tone, trace interval or shock periods (No effect of group *F*_(1,10)_ = 2.67, *p* > 0.05; Main effect of stimulus period *F*_(3,30)_ = 278.3 *p* < 0.05; No group × stimulus period interaction *F*_(3,30)_ = 1.59, *p* > 0.05; Figure 5A). Differences were also not observed when dCA1 was activated during these same periods *via* ChR2 stimulation (No effect of group *F*_(1,10)_ = 0.03, *p* > 0.05; Main effect of stimulus period *F*_(3,30)_ = 330.2, *p* < 0.05; No group × stimulus period interaction *F*_(3,30)_ = 0.31, *p* > 0.05; Figure 5B). These results are consistent with previous reports and indicate that stimulation or inhibition of dCA1 does not impair trace fear conditioning by inducing hyperactivity or preventing the animals from processing shock.

## Discussion

In this set of experiments, we compared the effects of optogenetic inhibition and stimulation of the dorsal hippocampus on the encoding and retrieval of trace fear memories. Our results demonstrate that intact dCA1 activity is required for the retrieval of both tone and context fear. This is true regardless of whether activity is decreased or increased. Although some previous work suggests that trace fear memories can be retrieved without the dorsal hippocampus (Yoon and Otto, 2007; Czerniawski et al., 2009; Cox et al., 2013), our results agree with previous studies that found lesions and pharmacological inactivation of this region impair trace fear expression (Chowdhury et al., 2005; Quinn et al., 2005; Raybuck and Lattal, 2011).

When dCA1 was inhibited during encoding, we found that tone fear memory was impaired, but memory for the training context remained intact. This is consistent with the fact that manipulations of the dorsal hippocampus during context fear learning often do not prevent memory formation (Maren et al., 1997; Frankland et al., 1998; Wiltgen et al., 2006). This finding is thought to reflect the ability of other brain areas (e.g., ventral hippocampus, prefrontal cortex) to compensate for the lack of dorsal hippocampus contributions to learning (Wiltgen and Fanselow, 2003; Rudy et al., 2004; Zelikowsky et al., 2013). In contrast, inactivation of the dorsal hippocampus after learning typically leads to robust retrograde amnesia for context fear (Kim and Fanselow, 1992; Maren et al., 1997; Anagnostaras et al., 1999; Matus-Amat et al., 2004), as seen in our retrieval experiments. Together, these data suggest that dCA1 is required for memory expression if this region is intact during learning (Moser and Moser, 1998; Wiltgen and Fanselow, 2003; Rudy et al., 2004).

Unlike inhibition, activation of dCA1 during training produced deficits in both tone and context fear memory. This more complete memory impairment suggests that the abnormal activity patterns induced by ChR2 stimulation disrupted encoding in brain regions that can normally compensate for the loss of the dorsal hippocampus. Consistent with this idea, stimulation of dCA1 has been shown to produce widespread increases in brain activity (Takata et al., 2015; Lebhardt et al., 2016). In contrast to our results, some studies have found that increases in CA1 activity during encoding enhance trace fear memory acquisition (Kitamura et al., 2014; Sellami et al., 2017). For example, Sellami et al. (2017) showed that direct stimulation of CA1 pyramidal cells during the trace interval attenuates trace fear conditioning deficits in aged mice. This discrepancy may be explained by differences in age between studies. Young mice show learning-related increases in CA1 intrinsic excitability following trace fear conditioning that are reduced with aging (Oh et al., 2010). It is possible that CA1 stimulation during the trace interval rescues this physiological impairment in old mice, ameliorating their trace fear conditioning deficits, but adds noise to the already-excitable hippocampus in younger animals. The effect of this noise on learning could be amplified by the higher stimulation frequency that was used in the current study (20 Hz vs. 5 Hz). Consistent with this idea, previous work showed that stimulation at 20 Hz could be used to induce freezing in the dentate gyrus but not in dCA1 (Ramirez et al., 2013). Our data are consistent with this observation and extend it by demonstrating that dCA1 stimulation at 20 Hz is actually disruptive to ongoing memory retrieval.

The current results support the idea that dCA1 is critically involved in forming and retrieving trace fear memories. Nonetheless, despite the extensive literature on this topic, the specific contribution of CA1 to these processes remains known. To better understand its role, future investigations will need to examine its unique physiological properties in more detail as well as characterize the type of information it receives from brain areas like the prefrontal cortex, entorhinal cortex and the nucleus reuniens.

## Data Availability

All datasets generated for this study are included in the manuscript.

## Ethics Statement

All experiments were reviewed and approved by the UC Davis Institutional Animal Care and Use Committee (IACUC).

## Author Contributions

JW and KP contributed equally to the design, implementation and analysis of experiments and to the writing of the manuscript. BW contributed to the design and analysis of experiments and to the writing of the manuscript.

## Conflict of Interest Statement

The authors declare that the research was conducted in the absence of any commercial or financial relationships that could be construed as a potential conflict of interest.
